# trumpet: transcriptome-guided quality assessment of m^6^A-seq data

**DOI:** 10.1186/s12859-018-2266-3

**Published:** 2018-07-13

**Authors:** Teng Zhang, Shao-Wu Zhang, Lin Zhang, Jia Meng

**Affiliations:** 1 Key Laboratory of Information Fusion Technology of Ministry of Education, School of Automation, Northwestern Polytechnical University, Xi’an, 710027 Shaanxi China; 2 School of Information and Control Engineering, China University of Mining and Technology, Xuzhou, 221116 Jiangsu China; 3 Department of Biological Sciences, Research Center for Precision Medicine, Xi’an Jiaotong-Liverpool University, Suzhou, 215123 Jiangsu China; 4 Institute of Integrative Biology, University of Liverpool, L7 8TX, Liverpool, UK

**Keywords:** m^6^A-seq, RNA methylation, Data quality, Assessment metrics, trumpet R package

## Abstract

**Background:**

Methylated RNA immunoprecipitation sequencing (MeRIP-seq or m^6^A-seq) has been extensively used for profiling transcriptome-wide distribution of RNA N6-Methyl-Adnosine methylation. However, due to the intrinsic properties of RNA molecules and the intricate procedures of this technique, m^6^A-seq data often suffer from various flaws. A convenient and comprehensive tool is needed to assess the quality of m^6^A-seq data to ensure that they are suitable for subsequent analysis.

**Results:**

From a technical perspective, m^6^A-seq can be considered as a combination of ChIP-seq and RNA-seq; hence, by effectively combing the data quality assessment metrics of the two techniques, we developed the trumpet R package for evaluation of m^6^A-seq data quality. The trumpet package takes the aligned BAM files from m^6^A-seq data together with the transcriptome information as the inputs to generate a quality assessment report in the HTML format.

**Conclusions:**

The trumpet R package makes a valuable tool for assessing the data quality of m^6^A-seq, and it is also applicable to other fragmented RNA immunoprecipitation sequencing techniques, including m^1^A-seq, CeU-Seq, Ψ-seq, etc.

**Electronic supplementary material:**

The online version of this article (10.1186/s12859-018-2266-3) contains supplementary material, which is available to authorized users.

## Background

Recent studies have shown that reversible N6-Methyl-Adnosine (m^6^A) RNA methylation plays important roles in regulating many cellular processes, including mRNA expression, splicing, translation, RNA-protein interaction, cell differentiation, etc. [1, 2]. Elucidating functions of RNA methylation is one the most active areas of research. Currently, the most widely used sequencing technology for profiling transcriptome-wide distribution of RNA methylation is MeRIP-seq or m^6^A-seq, which pulls down the RNA fragments that carry N6-Methyl-Adnosine modification with an anti-m^6^A antibody in the immunoprecipitation (IP) stage before sending them for sequencing [3, 4]; often, an input control sample is also generated to serve as the background control.

In recent years, m^6^A-seq has been widely applied to various species, such as, human, mouse, fly, zebrafish, rice and yeast, to uncover the functions of RNA m^6^A methylation. However, due to the chemical instability of RNA molecules and the intricate experiment procedures, special care is needed to ensure the quality of m^6^A-seq experiments, and often the data generated from m^6^A-seq technology may suffer from various defects, such as, DNA contamination, RNA degradation, and immunoprecipitation failure. Hence, assessing the quality of m^6^A-seq data is necessary to ensure that they are suitable for subsequent analysis.

Data quality assessment has been a critical issue for high-throughput sequencing technology in general, and a number of software tools have been developed for this purposes, including, e.g., FastQC for general sequencing data quality [5], RNA-SeQC and RseQC for RNA-seq data [6, 7], and CHANCE for ChIP-seq data [8]. However, due to the unique characteristics of m^6^A-seq data, neither of the aforementioned tools along is sufficient. To address this shortfall, we developed an R package, trumpet, which stands for transcriptome-guided q**u**ality assessment of **m**ethylated RNA immunopreci**p**itation s**e**quencing da**t**a. The trumpet package takes the aligned BAM files from m^6^A-seq data together with the transcriptome information as the inputs to generate a quality assessment report in the HTML format, which covers a number of metrics relevant to the m^6^A-seq data quality.

## Implementation

The trumpet R package takes the aligned BAM files of m^6^A-seq data together with the transcriptome annotation as the inputs, and returns an assessment report concerning the data quality with a single line of R command. The transcriptome annotation is necessary for separating the signal (transcribed regions) from the noise (non-transcribed regions), and may be provided as a GTF file or converted from other formats into a TxDb object [9]. This package supports the down-sampling of reads to ensure that the comparison is not affected by the different sequencing depths (library size) of the samples.

The quality of m^6^A-seq data is assessed by the trumpet package from mainly 3 perspectives, including (1) statistics of sequencing reads distribution with respect to different genomic regions; (2) the strength of the immunoprecipitation signal evaluated by the exome signal extraction scaling (ESES) and other statistical approaches; (3) comparison between different biological replicates to identify possible outliers. These assessment components are detailed in the following with a sample dataset that profiles midbrain gene under wild type and FTO knockdown conditions [10, 11]. The source code (see Additional file 1) and a comprehensive user’s manual are freely available at GitHub: https://github.com/skyhorsetomoon/Trumpet.

### Statistics of sequencing reads

This module is aimed to gain overall insights into samples via statistics of read counts, which is probably the most fundamental way to check the quality of samples. Relatively low number of reads or distinct proportion of reads mapped to a specific genomic region may be naturally associated to poor data quality due to unbalanced sequencing in sample multiplexing, DNA contamination or other bias during the experimental procedures. In this section, we mainly evaluate read alignment and their distribution, with which we inspect the sequencing depth of the input files, the heterogeneity of read coverage, the read alignment mapped to different genomic regions, such as exon, intron, 5’UTR, CDS and 3’UTR.

The Table 1 summarized the read alignment information from 6 m^6^A-seq samples, which profile the m^6^A epitranscriptome [12] in mouse midbrain under FTO knock-down [11]. It is observed that sample IP2 with GEO accession number GSM1147022 has less reads mapped to 3’UTR (29.0%) compared with the other samples (36.93, 34.97, 37.41, 36.27 and 37.63%), which may be due to the 3′ bias during sample preparation [13].

**Table 1:** Number of Reads Aligned to Different Genomic Regions

Sample ID	GEO	Total	Exon	Intron	Non-genic	5’UTR	CDS	3’UTR
IP1	GSM1147020	28.9 M	14.1 M (48.78%)	1.5 M (5.18%)	13.31 M (46.04%)	1.1 M (7.72%)	7.92 M (55.35%)	5.28 M (36.93%)
IP2	GSM1147022	11.6 M	5.71 M (49.18%)	0.6 M (5.17%)	5.3 M (45.65%)	0.52 M (9.68%)	3.3 M (61.42%)	1.56 M (29.0%)
IP3	GSM1147024	36.86 M	17.85 M (48.42%)	1.92 M (5.2%)	17.1 M (46.38%)	1.19 M (6.82%)	10.17 M (58.21%)	6.11 M (34.97%)
Input1	GSM1147021	14.82 M	6.52 M (43.99%)	0.47 M (3.17%)	7.83 M (52.84%)	0.31 M (7.43%)	2.3 M (55.16%)	1.56 M (37.41%)
Input2	GSM1147023	17.15 M	6.9 M (40.26%)	0.42 M (2.45%)	9.82 M (57.29%)	0.17 M (8.81%)	1.06 M (54.92%)	0.7 M (36.27%)
Input3	GSM1147025	18.15 M	7.63 M (42.06%)	0.46 M (2.54%)	10.05 M (55.4%)	0.28 M (7.37%)	2.09 M (55%)	1.43 M (37.63%)

The number of reads mapped to different regions is summarized as following. A summary table matching the sample ID with the input BAM files is also provided in the full report. Issues may be identified if a metrics is significantly different from other samples. E.g., the total number of reads and the reads mapped to 3’UTR of IP2 sample are both significantly different than all other IP samples

### Whole-transcriptome heterogeneity of read coverage

In order to show the heterogeneity of read coverage in the entire transcriptome due mainly to different levels of gene expression, PCR artifacts and randomness, we used bin-based approach to check the percentage of regions covered different number of reads. To make the result comparable and not affected by different sequencing depth, the same number of reads are randomly selected from each sample using the built-in option. In Table 2, IP1, IP2 and IP3 are three sample datasets from the mouse midbrain gene under FTO knockdown [11]. The sample IP2 has a higher percentage of reads in exonic regions and more regions covered with > 10^4^ reads compared with the other samples, suggesting a higher degree of heterogeneity in read coverage, which may indicate potential PCR artifacts during sample preparation or sequencing. This has been confirmed by the FASTQC [5] software, where the sample IP2 has highest Kmer content among the samples (fold enrichment of the most over-represented Kmer: 33.31 in IP2 vs 23.61 and 27.63 in IP1 and IP3). PCR artifacts may further exacerbate the existing heterogeneity in reads coverage of an m^6^A-seq experiment.

**Table 2:** Exonic Regions of Different Read Coverage

Sample ID	GEO	0	1~ 10	10^1^~ 10^2^	10^2^~ 10^3^	10^3^~ 10^4^	10^4^~ 10^5^
IP1	GSM1147020	11.77%	7.85%	25.45%	44.4%	10.34%	0.19%
IP2	GSM1147022	17.68%	8.91%	29.24%	35.55%	8.24%	0.38%
IP3	GSM1147024	11.15%	7.94%	26.01%	45.82%	8.94%	0.14%

### Visualization of reads distribution

**Fig. 1:**
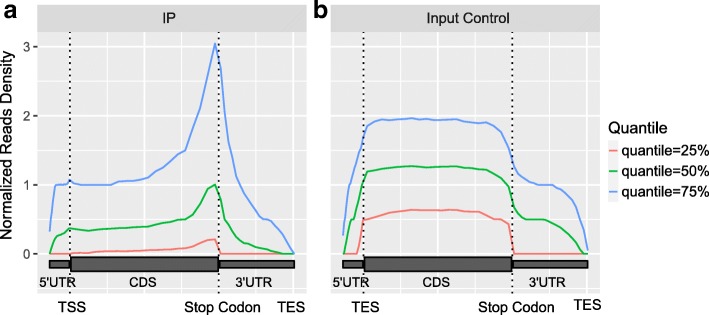
Distribution of reads. Figure shows that the reads are strongly enriched near stop codon in the IP sample (**a**) compared with the input sample (**b**), which is an expected pattern of the in an m^6^A-seq experiment. The enrichment is observed at all 3 different quantiles (25, 50 and 75%). The figure is plotted with metaPlotR R/Bioconductor package [34] via the trumpet package

It is known that, RNA m^6^A methylation is enriched near the stop codon. In this module, the distribution of reads in different genomic regions (5’UTR, CDS and 3’UTR) is visualized. Since there exist highly abundant genes, whose m^6^A enrichment signal may dominate the analysis if raw reads are directly used in the analysis, the same weight is assigned to all the detected genes regardless of their read coverage. Specifically, genes that are not expressed or have less than 10 reads are first excluded; then, for the remaining genes, the read coverages at different regions of the same transcript are counted and then standardized (divided by the read coverage’s mean counts). The quantiles (25, 50 and 75%) of the standardized read coverage at different genomic regions is then plotted as shown in Fig. 1.

### Assessing immunoprecipitation efficiency with ESES

A major aspect of the m^6^A-seq data quality is the efficiency of immunoprecipitation, which can be reflected by the enrichment of immunoprecipitation signal. To evaluate the enrichment of m^6^A signal in the IP sample, trumpet package uses a metric called exome signal extraction scaling (ESES), which is a modified form of the signal extraction scaling (SES) approach previously developed for assessing the signal of ChIP-seq data [8]. The ESES approach is different from the SES approach in two aspects. Firstly, the genome background of ChIP-seq data used in SES approach was replaced by standardized gene-specific exome background of MeRIP-seq data to exclude the influence of regions that do not carry meaningful signal (introns and non-genic regions). Secondly, the read coverage in MeRIP-seq data is normalized with respect to the expression level of that gene to eliminate the impact of different expression level of genes. More specifically, we first divide a gene into *n* bins and count the number of reads mapped to each bin. Let *y*_*t*, *g*, *i*_ be the read count of the *i*-th bin on the *g*-th gene in the IP sample, and*y*_*c*, *g*, *i*_ represent the read count of the *i*-th bin on the *g*-th gene in the Input sample. The standardized the read count that eliminates the difference in expression of genes can be calculated as

1 $$ {\overline{y}}_{t,g}=\frac{\sum_{\forall i}{y}_{t,g,i}}{n} $$

2 $$ {\overline{y}}_{c,g}=\frac{\sum_{\forall i}{y}_{c,g,i}}{n} $$

3 $$ {\widehat{y}}_{t,g,i}={y}_{t,g,i}/{\overline{y}}_{t,g} $$

4 $$ {\widehat{y}}_{c,g,i}={y}_{c,g,i}/{\overline{y}}_{c,g} $$

where ${\overline{y}}_{t,g}$ and ${\overline{y}}_{c,g}$ are the average number of reads mapped to each bin of the *g*-th gene in the IP and Input sample, respectively, which are proportional to the abundance of that gene and the sequencing depth (total number of reads) of sample, and ${\widehat{y}}_{t,g,i}$ and ${\widehat{y}}_{c,g,i}$are the enrichment signal and the background signal, respectively, which are normalized by ${\overline{y}}_{t,g}$ and ${\overline{y}}_{c,g}$, respectively. We then pool all the signal $\left\{{\widehat{y}}_{t,g,i}|\forall \left(g,i\right)\right\}$ together, and sort them in an increasing order to obtain a list of order statistics $\left\{{\widehat{y}}_{t,(i)}\right\}$, where ${\widehat{y}}_{t,(i)}$ denotes the *i*-th element of $\left\{{\widehat{y}}_{t,(i)}\right\}$, which is also the standardized read count of the bin with the *i*-th least number of the normalized reads mapped in $\left\{{\widehat{y}}_{t,g,i}|\forall \left(g,i\right)\right\}$. By this way, the bins that are enriched with m^6^A signals are likely to appear in the end of the list. If we assume that there are a total of *N* bins on the transcriptome surveyed in this analysis, then we should have (*i*) ∈ {1, 2, ⋯, *N*}. Meanwhile, let $\left\{{\widehat{y}}_{c,(i)}\right\}$ be the list of normalized read count of the merged Input sample that has been reordered to match $\left\{{\widehat{y}}_{t,(i)}\right\}$, i.e., let $\left\{{\widehat{y}}_{c,(i)}\right\}$ and $\left\{{\widehat{y}}_{t,(i)}\right\}$ denote the normalized read count of the same ordinal bin in the Input and IP sample, respectively. The following procedures are similar to the original SES metrics. We denote the cumulative summation of $\left\{{\widehat{y}}_{t,(i)}\right\}$ and $\left\{{\widehat{y}}_{c,(i)}\right\}$ by.

5 $$ {y}_t(j)=\sum \limits_{i=1}^j{\widehat{y}}_{t,(i)} $$

6 $$ {y}_c(j)=\sum \limits_{i=1}^j{\widehat{y}}_{c,(i)} $$

**Fig. 2:**
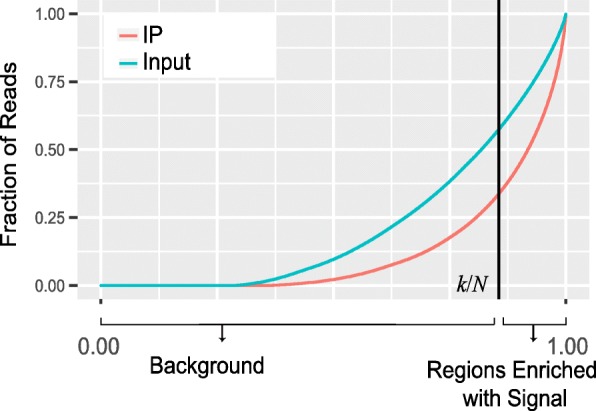
Quality assessment of immunoprecipitation signal with ESES. In this figure, the black straight line divides IP sample into two parts: the background (the left-hand side) and the region enriched with immunoprecipitation signal (the right-hand side). The fraction of cumulated signal (Faction of Reads) shows the fraction of reads captured in the corresponding regions, and the difference in cumulated signals between the IP and Input samples (or the scale factor) is shown by the two cross-points between the two curves (IP curve and Input curve) and the black straight line. Please refer to section “Assessing immunoprecipitation efficiency with ESES” for more details. Similar to before, we consider here only the genes that have at least 10 mapped reads. Transcripts with less 10 reads mapped are considered not readily detected and thus are excluded from this analysis

If we consider a total of *N* bins on the transcriptome surveyed in this analysis, it is then possible to calculate a fraction of cumulative immunoprecipitation signal in the IP sample as *p*_*j*_ = *y*_*t*_(*j*)/*y*_*t*_(*N*) and also the cumulative background information in the input sample as*q*_*j*_ = *y*_*c*_(*j*)/*y*_*c*_(*N*). Because the bins are arranged in an increasing order of normalized read count, the bins that are enriched with m^6^A signal are likely to appear in the very end of the list. For this reason, as *j* increases from 1 to *N*, *p*_*j*_ should first increases slower than *q*_*j*_ before reaching bins absent of m^6^A and then increases faster than *q*_*j*_ afterwards. Moreover, ∣*q*_*j*_ − *p*_*j*_∣, which computes the difference in the cumulative percentage between IP and Input samples, will also first increase from 0 with as *j* increases but decrease rapidly once the bins with sufficiently large read count or enriched signals are incorporated (see Fig. 2). Consistent with the SES approach, the background component in the IP data can be obtained by identifying the locations of the bin with*k* = max_*j*_ ∣ *q*_*j*_ − *p*_*j*_∣, where the fraction allocation of reads in the unified Input sample maximally exceeds that of the IP sample. The first *k* bins are then identified as the background region of IP sample and the bins afterwards are defined as the regions enriched with the immunoprecipitation signal in IP sample. We then define the fraction of regions enriched with signal (*k*/*N*) and also the scale factor (max_*j*_ ∣ *q*_*j*_ − *p*_*j*_∣) to show the degree of difference between the IP and Input samples.

The reported ESES metrics on the sample dataset is shown in Table 3, from which we can see that the IP2 sample is substantially different from the others with a much smaller region enriched with signal and a much larger scale factor.

**Table 3:** ESES metrics from the sample dataset

Sample ID	GEO	Percent of Region Enriched with Signal	Scale Factor
IP1	GSM1147020	13.23%	0.24
IP2	GSM1147022	11.93%	0.4
IP3	GSM1147024	13.62%	0.22

We can see that the second IP sample (IP2) is substantially different from the other samples, which is consistent with the previous results. Reads are down-sampled to 10 million for a fair comparison among all the samples

### Assessing the enrichment of m^6^A signal with C-test

**Fig. 3:**
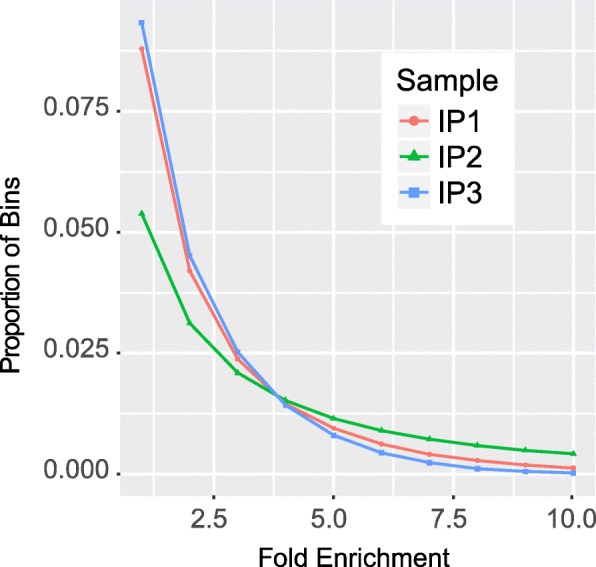
Assessing the enrichment of m^6^A signal with C-test. Figure shows that around 2.5% of regions are enriched with reads in the IP samples with fold enrichment larger than 2.5 and a major difference between the IP 2 sample and the other samples is observed in their respective enrichment profiles, which is consistent with the previous analysis (see Tables 1, 2 and 3)

Besides the ESES metrics, the trumpet package also include a C-test to detect the regions enriched with m^6^A signals at different levels. The C-test compares two Poisson means and is used in the exomePeak package to predict RNA methylation sites [14]. This is a more straightforward measurement to elucidate the statistical difference of the IP and Input samples. Specifically, only the bins that overlap with more than 10 reads are considered in the analysis, and the proportion of bins that are enriched in the IP sample with m^6^A signal at different fold enrichment thresholds are counted and plotted. It is then possible to compare the difference between different samples. As shown in Fig. 3, the C-test detected a major difference between IP2 and the others, which is consistent with the previous analysis.

### Hierarchical clustering and PCA analysis of samples

**Fig. 4:**
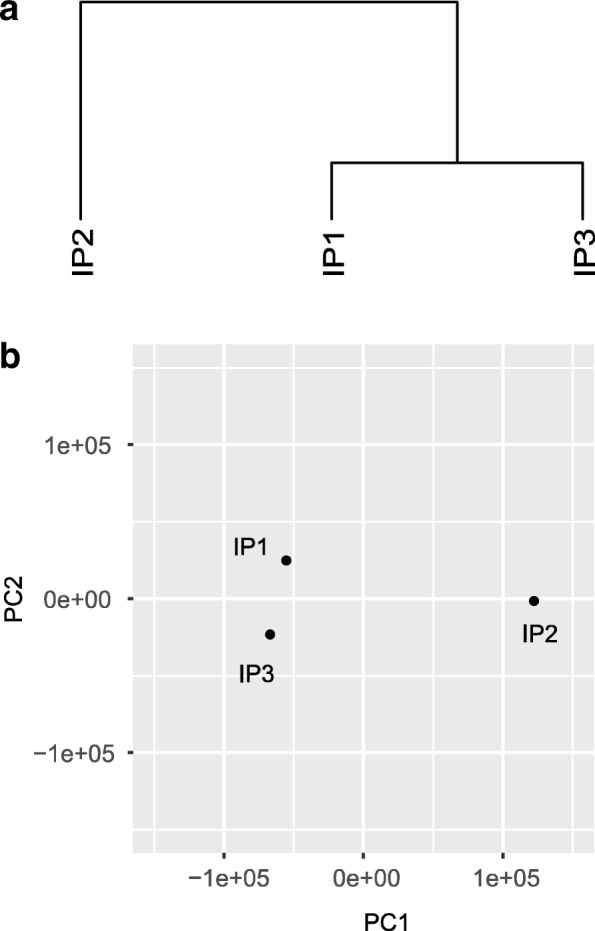
Hierarchical clustering and PCA analysis of the samples. Hierarchical clustering and PCA analysis is performed to show relative similarity of the samples. As shown in Fig. 4, the IP2 sample is more different from the other samples based on both hierarchical clustering analysis (**a**) and PCA analysis (**b**). The analysis of samples were performed based on the normalized reads counts of all the bins in the transcriptome in R. Specifically, the hierarchical clustering is implemented with hclust command based on Euclidean distance and the default setting; while the PCA analysis is implemented with the prcomp function in the stats R package

Hierarchical Clustering (HC) is then applied to all the samples for the identification of possible outliers and for assessing the relative similarity between samples and groups (if applicable). To eliminate the impact of different sequencing depth and transcriptional regulation, the hierarchical clustering is performed as follows. Let *x*_*i*, *j*_ represent the number of reads of the *i*-th bin located on the exome in the *j*-th sample. The standardized read count after eliminating difference in sequencing depth can be calculated as

$$ {y}_j=\sum \limits_i^N{x}_{i,j} $$

$$ {s}_j=\frac{y_j}{\exp \left[\frac{1}{N}\sum \limits_{n=1}^N\log \left({y}_n\right)\right]} $$

$$ {\tilde{y}}_{i,j}=\frac{y_{i,j}}{s_j} $$

where *y*_*j*_ is the total number of reads in the *j*-th sample, *s*_*j*_ is the size factor of the *j*-th sample, and ${\tilde{y}}_{i,j}$is the standardized reads count of the *i*-th bin in the *j*-th sample. We can then perform HC and PCA analysis to study the relationship between different samples, as shown in Fig. 4. Please note that this is a bin-based analysis, where the association between bins and genes is not used.

### Gene-specific heterogeneity of read coverage

**Fig. 5:**
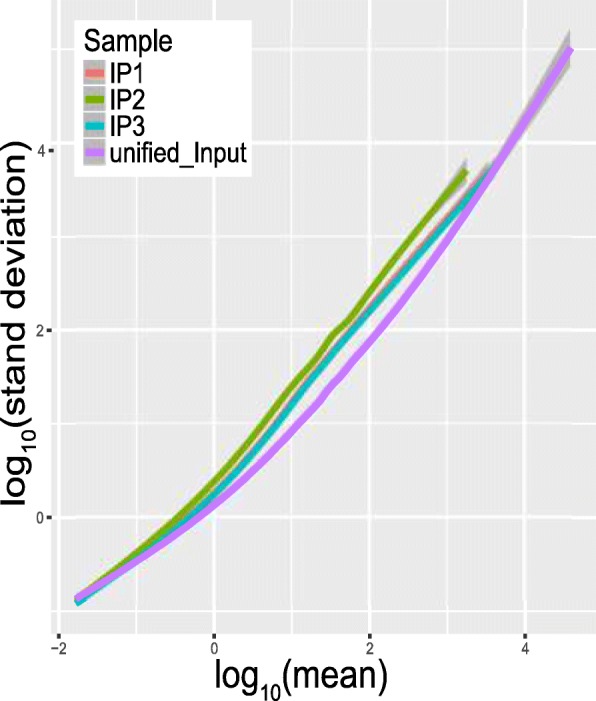
Gene-specific heterogeneity of read coverage. The IP2 sample has the largest heterogeneity in read coverage, while the other two IP samples are quite similar. This is consistent with our previous results, which suggest that IP2 sample may be problematic (see Tables 1, 2 and 3 and Figs. 3 and 4)

In practice, the aligned reads are not evenly distributed on the same gene. In the IP sample, heterogeneity of read coverage may be generated from the enrichment signal around the true methylation sites, which may be complicated due to isoform ambiguity, or some possible bias and artifacts due to PCR process. Compared with the IP sample, the coverage is more flat in the input sample. The gene-specific heterogeneity of read coverage is assessed in the trumpet package with the mean and standard deviation (SD) of read count in each gene of each sample. Specifically, let *y*_*j*, *g*, *i*_ be the read count of the *i*-th bin on the *g*-th gene in the *j* -th IP sample, and ${\overline{y}}_{j,g}$ and *SD*_*j*, *g*_represent the average number of reads mapped to the bins on the*g*-th gene in the *j*-th IP sample and its standard deviation. We then use a local regression to fit a curve between ${\overline{y}}_{j,g}$ and *SD*_*j*, *g*_ for ∀*g*. As shown in Fig. 5, the IP2 sample has the largest heterogeneity in the read coverage, suggesting that it is quite different from the other samples, which is consistent with our previous results. Replicates are usually expected to exhibit similar patterns, and this is especially true if the pattern is a robust pattern obtained by summarizing from signals in the entire transcriptome. If a sample is quite different from the other replicates, it is probably worthwhile to investigate the cause of it. Additionally, compared with the unified input sample (generated by merging all the input samples under that condition), the IP samples should have much larger heterogeneity, because the IP samples contain additional enrichment signals and are generated with a more complex procedure that may introduce additional noise.

### Sample consistency and reproducibility

This metric is used to assess the degree of difference between multiple biological replicates. In order to eliminate the difference in sequencing depth between theses biological replicates, we first normalize the read count of the each bin in each sample. The normalization method is the same as the hierarchical clustering analysis detailed in Section “Hierarchical clustering and PCA analysis of samples”. Let ${\tilde{y}}_{i,j}$ be the standardized read counts of the *i*-th bin in the *j*-th sample. Also assume that we have multiple biological replicates (*j* ∈ {1, 2, ⋯, *J*}) obtained from the same experimental condition. Then, the mean and standard deviation of the read counts of the same bin across different samples can be calculated as:

$$ {\mu}_i=\frac{1}{J}\sum \limits_{j=1}^J{\tilde{y}}_{i,j} $$

$$ {s}_i=\sqrt{\sum_{j=1}^J{\left({\tilde{y}}_{i,j}-{\mu}_i\right)}^2}\frac{1}{\sqrt{J-1}} $$

It is possible to fit the variables with a local regression curves to show the consistency between different samples, or compare the reproducibility of the samples obtained from different conditions.

## Results

We included in the following 4 case studies to show that: (**a**) There exists increased variability in the RNA methylation level due to gene knock down operation; (**b**) Different immunoprecipitation efficiency is observed on datasets using antibodies from different companies, (**c**) The RNA m^6^A methylation level of U2OS cell line is relatively high compared with other cell line, (**d**) m^6^A-seq is enriched near stop codon while m^1^A is enriched on 5’UTR.

### Gene knock down induces additional variability among replicates

**Fig. 6:**
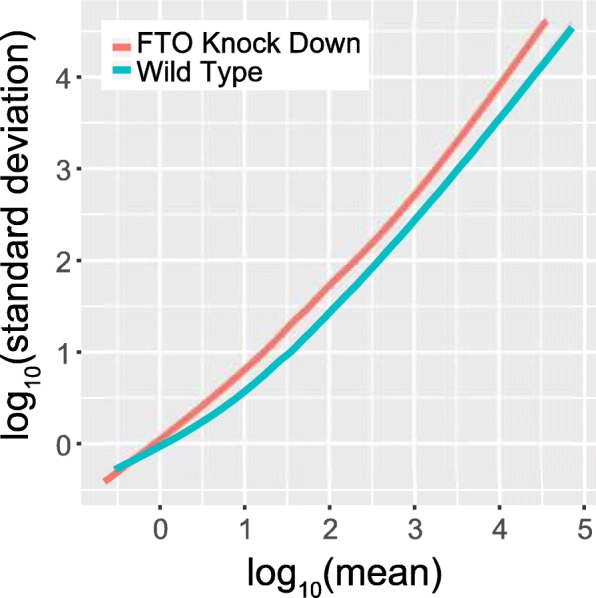
Comparing the sample reproducibility. The samples obtained under wild type condition shows obvious higher reproducibility compared with those obtained under FTO knock down condition, which requires additional operations that may account for the increased variability in samples

In this example, we compared the m^6^A-seq samples obtained under wild type and FTO knock down condition [10, 11] in terms of sample consistency and reproducibility (see Section “Sample consistency and reproducibility”). As shown in Fig. 6, the samples obtained under FTO knock down condition show higher within-group variability compared with those obtained under the wild-type condition, suggesting the FTO knock down process induced additional variability among the samples. Direct comparison of two groups of samples is supported by trumpet package.

### Failure to capture enrichment signal in lowly expressed genes

**Fig. 7:**
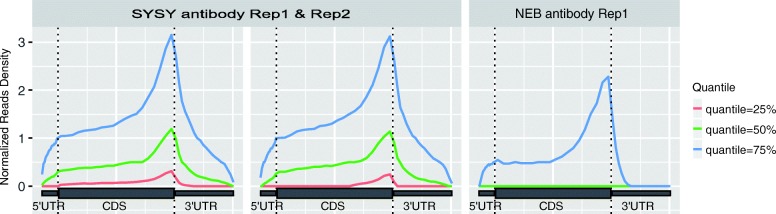
Reads distribution in samples using antibody from different companies. The m^6^A signal can still be readily observed on lowly expressed genes (corresponding to 25 and 50% quantile green and red curves) in the two SYSY samples, but not in the NEB sample, suggesting NEB sample suffers from some artifacts

In this example, we considered reads distributions of the m^6^A-seq samples profiling the m^6^A epitranscriptome in HEK293T using anti-m^6^A antibodies made from different companies (SYSY and NEB) [4] (see section “Visualization of reads distribution”). In order to eliminate the impact of different sequencing depth of the samples, down-sampling of the m^6^A-seq samples to 10 million mapped reads was first performed before further analysis. As shown in Fig. 7, the enrichment of reads near the stop codon can be observed for the highly expressed genes (corresponding to 75% quantile blue curve) in all 3 samples, suggesting that all 3 samples no matter which the antibody was made captured the m^6^A signal. The m^6^A signal can still be readily observed on relatively lowly expressed genes (corresponding to 25 and 50% quantile green and red curves) in the two SYSY samples, but not in the NEB sample, suggesting NEB sample suffers from potential artifacts. One possible explanation is that there was insufficient amount of RNA in the NEB sample. Because the input material did not contain a large variety of RNAs, the sequencing data thus failed to capture the m^6^A signal in lowly expressed genes. Please note that all the samples here were from the same study, were generated by the same protocol, had the same number of reads after down-sampling, and profiled the same cell type (HEK293T).

### Comparison of the m^6^A signal in different cell types

**Fig. 8:**
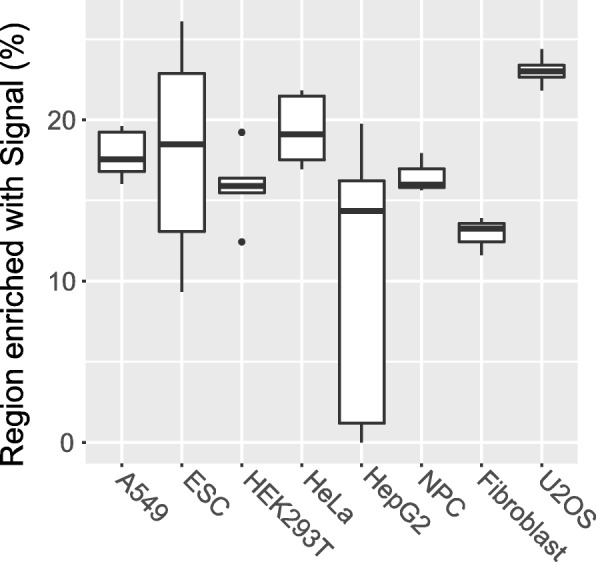
The percentage of regions enriched with m^6^A signal in different cell types. U2OS cell line has the highest proportion of regions enriched with m^6^A signal, while Fibroblast has the lowest

In this example, we compared the m^6^A signal detected in different cell types. The raw data was downloaded from published studies [4, 15–18] profiling the m^6^A epitranscriptome in different cell types, including A549, embryo stem cell (ESC), HEK293T, HeLa, neural progenitor cells (NPC), fibroblasts and U2OS. Under the default setting of the trumpet R package, the U2OS cell line is reported to have the largest percentage of regions enriched with m^6^A signal, suggesting the m^6^A methylation level is relatively high in this cell line, as shown in Fig. 8.

### Assessing m^1^A epitranscriptome sequencing data

Besides m^6^A-seq data, the trumpet package can also be applied to other affinity-based fragmented RNA immunoprecipitation sequencing data, such as m^1^A-seq [19] and PSU-seq [20]. As an example, we applied trumpet package to the m^1^A-seq dataset profiling the m^1^A epitranscriptome in HEK293T cell line [19], and compared this dataset with the m^6^A-seq data obtained from the same cell line [4].

As shown in Table 4, there exists distinct difference between m^1^A-seq and m^6^A-seq, the less abundant m^1^A modification is enriched in 7–8% of regions, while the more abundant m^6^A modification is enriched in 14.5% of region. The scale factor of the m^1^A-seq samples are a lot larger compared with that of m^6^A-seq.

**Table 4:** Comparison of m^1^A-seq and m^6^A-seq with ESES metrics

Modification Type	Technique	Sample ID	Region enriched with Signal	Scale Factor
m^1^A	m^1^A-seq	Rep1	7.01%	0.66
		Rep2	8.62%	0.6
m^6^A	m^6^A-seq	Rep1	14.48%	0.1

**Fig. 9:**
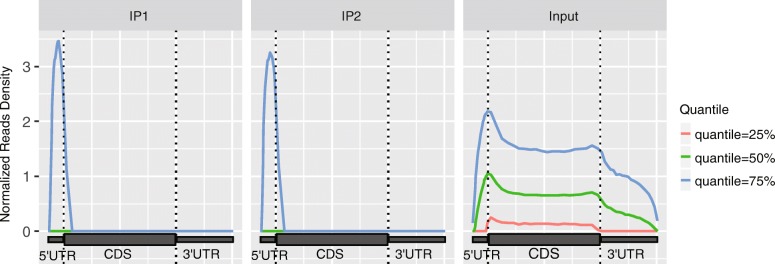
The reads distribution on m^1^A-seq data. The reads in the IP sample of m^1^A-seq data is enriched at 5’UTR, consistent with the distribution of m^1^A modification. The reads in the Input control sample are relatively evenly distributed and slightly enriched near start codon and stop codon. It is unclear whether this pattern is related to the experiment protocol

Additionally, the reads in the IP sample of m^1^A-seq data is enriched in the 5’UTR, which is consistent with the known distribution of m^1^A modification. However, similar to case study 2, m^1^A signal was not observed for very lowly expressed genes (see Fig. 9).

### Typical metric values obtained on published datasets

**Fig. 10:**
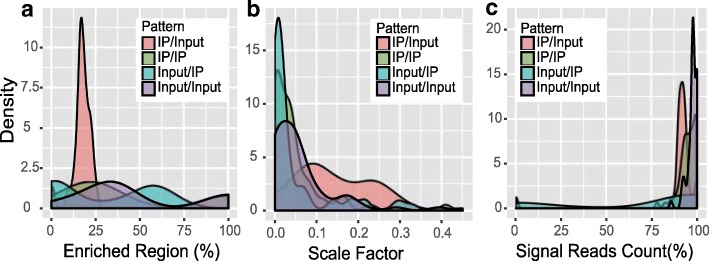
Reasonable ranges of metrics. The 3 major metrics, i.e., the percentage of enrichment region, scale factor and percentage of signal read counts, are calculated on data from recent high impact studies, representing data of reasonable good quality (IP/Input) and from data of poor quality generated by sample swop. Specifically, the Input samples are replaced with IP samples from other condition in IP/IP group, the IP samples are replaced by Input samples from other condition in Input/IP group, and the Input and IP samples are swopped in Input/Input group. The datasets of reasonable quality shows quite different pattern compared with other groups. The reasonable ranges of the enriched region, scale factor and signal reads count are 12%~ 25%, 0.08~ 0.3 and 87%~ 95%, respectively

The trumpet package reports a few metrics related to the quality of m^6^A-seq data, including notably, the scale factor, enriched regions, and signal read counts. However, due to the lack of a gold standard dataset and the variable m^6^A methylation level in different cell types, tissues and conditions, it is difficult to assert whether a dataset is of reasonable quality even provided with those metrics. To provide a global assessment of the m^6^A data quality, we collected 61 m^6^A-seq IP samples together with 59 corresponding Input samples from recent high impact studies [4, 15–18] and calculated these metrics as the positive control for reference; meanwhile, a number of m^6^A-seq samples of questionable data quality are generated by sample swapping, i.e., treating IP samples as input samples, or input samples as IP samples. As shown in Fig. 10, the metrics obtained on data of good quality (IP/Input) shows distinct pattern compared with those of poor quality (generated by sample swapping, i.e., IP/IP, Input/IP, and Input/Input). The ranges of these metrics for good quality samples are as follows; enriched region:12%~ 25%), scale factor: 0.08~ 0.3, and signal reads count: 87%~ 95%.

## Conclusion

An open source R package is developed for m^6^A-seq data quality control. With detailed documentation and the metrics of 61 datasets obtained from existing studies, it is possible to evaluate whether a new dataset is of reasonable quality. Although originally developed for the quality assessment of m^6^A-seq data, the trumpet package is equally applicable to other fragmented RNA immunoprecipitation sequencing techniques [14], including m^1^A-seq [19], CeU-Seq [21], Ψ-seq [22] and hMeRIP-seq [17], and may facilitate various epitranscriptome analysis, such as, site detection [23], differential methylation [24, 25], epitranscriptome module detection [26–28], network-based analysis [29], etc. [30, 31]. Nevertheless, it is worth mentioning that there are some general data quality metrics not covered by trumpet, e.g., reads quality, PCR artifacts, adaptor contamination, GC bias, etc., which should be assessed in the data analysis pipeline by other existing quality assurance software tools, such as, FastQC [5] and Qualimap [32].

Additionally, the gene annotation required by the trumpet package may still affect the metrics. Larger gene annotation databases may report more reads aligned to the exonic regions compared with smaller gene annotation database, and the ESES enrichment metrics can also be slightly affected (see Additional file 2: **Table S1-S4**). It is important to use similar gene annotation database for comparison purpose. By default, the UCSC gene annotation can be downloaded automatically from the internet from the trumpet package. Since the majority m^6^A-seq samples are constructed from polyA selected RNA libraries, only the exonic signals mapped to mRNA will be used, and the intronic signals are discarded from the analysis. In case it is desirable to analyze intronic signals or the RNA methylation on pre-mRNA, the library should be constructed from ribo-minus RNA library, and a gene annotation database including pre-mRNA should be constructed and provided to trumpet. From a computational perspective, pre-mRNA is no difference from an isoform transcript; however, the analysis performed will be affected by added transcriptome complexity and additional intronic regions with relatively lower signal-to-noise ratio. Another concern is from the rRNA. In theory, rRNA should not exist in the m^6^A-seq library when the two popular protocols, i.e., polyA selected or ribo-minus library, are used, and as a result, the trumpet package did not specifically test the impact of rRNA annotations on the m^6^A-seq data quality. As shown in Additional file 2: **Table S1-S3**, the amount of rRNA in m^6^A-seq data is likely to be very small; however, in practice, it may be still possible to see m^6^A-seq data targeting rRNAs, because the existence of RNA modifications on rRNA has been well established and their functions are often of interests [33]. The trumpet package should be used with extra caution in such cases because when the rRNA is highly abundant and it would dominate the evaluation metrics.

Due to a lack of gold standard m^6^A-seq datasets, we can only assess the relative data quality by comparing among samples without being able to determine with certain the true quality of data. It is thus necessary to generate gold standard datasets with carefully designed experiments or from using higher precision and higher resolution alternative technology such as miCLIP.

## Additional files

Additional file 1: Source code of the trumpet R package. (ZIP 2229 kb)

Additional file 2: Supplementary Material (including **Table S1-S4**) for trumept. (DOCX 21 kb)

## Abbreviations

hMeRIP-seq: Hydroxymethylcytosine sequencing
IP: The immunoprecipitation
m^1^A: N1-methyladenosine
m^6^A: N6-methyladenosine
MeRIP-Seq: methylated RNA immunoprecipitation sequencing
trumpet: transcriptome-guided quality assessment of methylated RNA immunoprecipitation sequencing data
Ψ-seq: Pseudouridine sequencing
