# Supplementary material for: trumpet: transcriptome-guided quality assessment of m6A-seq data
Source: BMC Bioinformatics. 2018 Jul 13;19:260. doi: 10.1186/s12859-018-2266-3 (PMC6044007; doi:10.1186/s12859-018-2266-3)
Supplement: Supplementary file 1 — Source code of the trumpet R package. (ZIP 2229 kb) [file 12859_2018_2266_MOESM1_ESM.zip › Trumpet-master/inst/doc/Trumpet-Overview.pdf]

An Introduction to *Trumpet* Package

Teng Zhang <tengzhagn156@126.com>

Modified: 2 April, 2017. Compiled: June 20, 2018

1 Quick Start with Trumpet

Trumpet stands for **tr**anscriptome-guided q**u**ality assessment of **m**ethylated RNA immunopreci**p**itation sequencing da**t**a. Trumpet is designed for the quality assessment of methylated RNA immunoprecipitation sequencing data.

The Trumpet package takes the aligned BAM files from m6A-seq data together with the annotation information as the input to generate a quality assessment report in HTML format, which covers a number of metrics that are relevant to the quality of m6A-seq data. The following R scripts show how to use this package to generate the assessment report.

```
> library(Trumpet)
> # Input the path of all the aligned MeRIP-seq data files in BAM format.
>
> f1 <- system.file("extdata", "IP1.bam", package="Trumpet")
> f2 <- system.file("extdata", "IP2.bam", package="Trumpet")
> f3 <- system.file("extdata", "IP3.bam", package="Trumpet")
> f4 <- system.file("extdata", "IP4.bam", package="Trumpet")
> f5 <- system.file("extdata", "Input1.bam", package="Trumpet")
> f6 <- system.file("extdata", "Input2.bam", package="Trumpet")
> f7 <- system.file("extdata", "Input3.bam", package="Trumpet")
> f8 <- system.file("extdata", "treated_IP1.bam", package="Trumpet")
> f9 <- system.file("extdata", "treated_Input1.bam", package="Trumpet")
> ip_bam <- c(f1,f2,f3,f4)
> input_bam <- c(f5,f6,f7)
> ip_bam_treated <- c(f8)
> input_bam_treated <- c(f9)
```

We then need to provide the transcriptome annotation with a `GTF`, a `TXDB` object, or let the package download this information from UCSC automatically.

```
> # We use GTF file as the transcriptome annotation information in the following example.
>
> gtf <- system.file("extdata", "hg19toy.gtf", package="Trumpet")
```

After input the MeRIP-seq data and annotation inforamtion file, we can call the main function to generate the assessment report in HTML format.

```
> trumpet_report <- Trumpet_report(IP_BAM = ip_bam,
+                                   Input_BAM = input_bam,
+                                   contrast_IP_BAM = contrast_ip_bam,
+                                   contrast_Input_BAM = contrast_input_bam,
+                                   condition1 = "untreated",
+                                   condition2 = "treated",
+                                   GENE_ANNO_GTF = gtf)
> # An HTML report will be generated under the current working directory.
> # And it can be opened with a web browser or with the following R command.
>
> browseURL("Trumpet_report.html")
```

2 Session Information

```
> sessionInfo()

R version 3.4.3 (2017-11-30)
Platform: x86_64-w64-mingw32/x64 (64-bit)
Running under: Windows 7 x64 (build 7601) Service Pack 1

Matrix products: default

locale:
[1] LC_COLLATE=Chinese (Simplified)_People's Republic of China.936
[2] LC_CTYPE=Chinese (Simplified)_People's Republic of China.936
[3] LC_MONETARY=Chinese (Simplified)_People's Republic of China.936
[4] LC_NUMERIC=C
[5] LC_TIME=Chinese (Simplified)_People's Republic of China.936

attached base packages:
[1] stats     graphics  grDevices utils     datasets
[6] methods   base

loaded via a namespace (and not attached):
[1] compiler_3.4.3 tools_3.4.3
```
