# Supplementary material for: trumpet: transcriptome-guided quality assessment of m6A-seq data
Source: BMC Bioinformatics. 2018 Jul 13;19:260. doi: 10.1186/s12859-018-2266-3 (PMC6044007; doi:10.1186/s12859-018-2266-3)
Supplement: Supplementary file 2 — Supplementary Material (including Table S1-S4) for trumept. (DOCX 21 kb) [file 12859_2018_2266_MOESM2_ESM.docx]

Additional files

**Trumpet: transcriptome-guided quality assessment of m^6^A-seq data**

**Table S1 Summary of Reads Alignment based on Ensemble Annotation**

| **Sample ID** | **Total** | **Exon** | **Intron** | **Non-genic** | **5’UTR** | **CDS** | **3’UTR** | **rRNA** |
| --- | --- | --- | --- | --- | --- | --- | --- | --- |
| **IP1 (**GSM928399**)** | 88.8M | 37.89M (42.68%) | 1.86M (20.94%) | 3.23M (36.38%) | 4.66M (12.4%) | 20.38M (54.25%) | 12.53M (33.35%) | 380 |
| **IP2 (**GSM928401**)** | 61.1M | 25.17M (41.2%) | 12.19M (19.95%) | 23.74M (38.85%) | 2.18M (8.88%) | 15.47M (63.04%) | 6.89M (28.08%) | 165 |
| **Refer_IP1**  **(**GSM928403**)** | 33.87M | 14.61M (43.15%) | 6.89M (20.35%) | 12.36M (36.5%) | 1.13M (7.78%) | 8.56M (58.95%) | 4.83M (33.27%) | 385 |

**Table S2 Summary of Reads Alignment based on UCSC Annotation**

| **Sample ID** | **Total** | **Exon** | **Intron** | **Non-genic** | **5’UTR** | **CDS** | **3’UTR** | **rRNA** |
| --- | --- | --- | --- | --- | --- | --- | --- | --- |
| **IP1 (**GSM928399**)** | 88.8M | 35.07M (39.5%) | 14.72M (16.58%) | 39M (43.92%) | 4.76M (14.25%) | 18.83M (56.32%) | 9.84M (29.43%) | 380 |
| **IP2 (**GSM928401**)** | 61.11M | 23.1M (37.81%) | 11.74M (19.28%) | 26.22M (42.91%) | 2.28M (12.3%) | 10.89M (58.78%) | 5.36M (28.92%) | 165 |
| **Refer_IP1**  **(**GSM928403**)** | 33.87M | 14.25M (42.07%) | 5M (14.76%) | 14.62M (43.17%) | 1.18M (8.58%) | 8.56M (62.13%) | 4.03M (29.29%) | 385 |

**Table S3 Summary of Reads Alignment based on RefSeq Annotation**

| **Sample ID** | **Total** | **Exon** | **Intron** | **Non-genic** | **5’UTR** | **CDS** | **3’UTR** | **rRNA** |
| --- | --- | --- | --- | --- | --- | --- | --- | --- |
| **IP1 (**GSM928399**)** | 88.8M | 31.19M (42.68%) | 11.3M (12.72%) | 46.31M (52.15%) | 3.05M (9.96%) | 18.32M (59.85%) | 9.24M (30.19%) | 380 |
| **IP2 (**GSM928401**)** | 61.11M | 17.4M (28.48%) | 7.5M (12.27%) | 36.21M (59.25%) | 1.58M (9.22%) | 10.54M (61.53%) | 5.01M (29.25%) | 165 |
| **Refer_IP1**  **(**GSM928403**)** | 33.87M | 13.08M (38.61%) | 4.08M (12.05%) | 16.71M (49.34%) | 0.81M (6.53%) | 7.84M (63.28%) | 3.74M (30.19%) | 385 |

**Table S4 ESES metrics based on Different Gene Annotations**

|  | **Gene Annotation Used** | | | | | |
| --- | --- | --- | --- | --- | --- | --- |
|  | **Ensemble** | | **UCSC** | | **RefSeq** | |
| **Sample ID** | Region  w/ Signal | Scale  Factor | Region  w/ Signal | Scale  Factor | Region  w/ Signal | Scale  Factor |
| **IP1 (**GSM928399**)** | 16.97% | 0.22 | 16.71% | 0.24 | 16.09% | 0.25 |
| **IP2 (**GSM928401**)** | 16.88% | 0.24 | 16.38% | 0.25 | 15.87% | 0.26 |
| **Refer_IP1 (**GSM928403**)** | 12.97% | 0.4 | 12.79% | 0.44 | 12.29% | 0.45 |
